# Dream Generation and Recall in Daytime NREM Sleep of Patients With Narcolepsy Type 1

**DOI:** 10.3389/fnins.2020.608757

**Published:** 2020-11-27

**Authors:** Carlo Cipolli, Fabio Pizza, Claudia Bellucci, Michela Mazzetti, Giovanni Tuozzi, Stefano Vandi, Giuseppe Plazzi

**Affiliations:** ^1^Department of Specialty, Diagnostic and Experimental Medicine, University of Bologna, Bologna, Italy; ^2^Department of Biomedical and Neuromotor Sciences, University of Bologna, Bologna, Italy; ^3^Istituto di Ricovero e Cura a Carattere Scientifico “Istituto delle Scienze Neurologiche” di Bologna, Bologna, Italy; ^4^Department of Psychology, University of Bologna, Bologna, Italy; ^5^Department of Biomedical, Metabolic and Neural Sciences, University of Modena and Reggio Emilia, Modena, Italy

**Keywords:** dream experience, narcolepsy type 1, multiple sleep latency test, SOREMP sleep, NREM sleep, dream recall

## Abstract

The less rigid architecture of sleep in patients with narcolepsy type 1 (NT1) compared with healthy subjects may provide new insights into some unresolved issues of dream experience (DE), under the assumption that their DE frequencies are comparable. The multiple transition from wakefulness to REM sleep (sleep onset REM period: SOREMP) during the five trials of the Multiple Sleep Latency Test (MSLT) appears of particular interest. In MSLT studies, NT1 patients reported a DE after about 80% of SOREMP naps (as often as after nighttime REM sleep of themselves and healthy subjects), but only after about 30% of NREM naps compared to 60% of daytime and nighttime NREM sleep of healthy subjects. To estimate accurately the “real” DE frequency, we asked participants to report DE (“dream”) after each MSLT nap and, in case of failure, to specify if they were unable to retrieve any content (“white dream”) or DE did not occur (“no-dream”). The proportions of dreams, white dreams, and no dreams and the indicators of structural organization of DEs reported after NREM naps by 17 adult NT1 patients were compared with those reported by 25 subjects with subjective complaints of excessive daytime sleepiness (sc-EDS), who take multiple daytime NREM naps. Findings were consistent with the hypothesis of a failure in recall after awakening rather than in generation during sleep: white dreams were more frequent in NT1 patients than in sc-EDS subjects (42.86 vs 17.64%), while their frequency of dreams plus white dreams were similar (67.86 and 61.78%) and comparable with that of NREM-DEs in healthy subjects. The longer and more complex NREM-DEs of NT1 patients compared with sc-EDS subjects suggest that the difficulty in DE reporting depends on their negative attitude toward recall of contents less vivid and bizarre than those they usually retrieve after daytime SOREMP and nighttime REM sleep. As this attitude may be reversed by some recall training before MSLT, collecting wider amounts of DE reports after NREM naps would cast light on both the across-stage continuity in the functioning of cognitive processes underlying DE and the difference in content and structural organization of SOREM-DEs preceded by N1 or also N2 sleep.

## Introduction

Dream experience (DE) of patients with chronically altered sleep organization may provide important insights into how neurophysiological and psychological processes interact in its generation (for review, see Schredl, 2009). DE of patients with narcolepsy type 1 (NT1, i.e., with cataplexy), which is pathophysiologically linked to the loss of the hypothalamic neurons producing hypocretin (American Academy of Sleep Medicine [AASM], 2014), is of potentially high theoretical interest, notwithstanding the low prevalence rate of this brain disease (ranging from 25 to 50 out of 100,000 people: Bassetti et al., 2019). Indeed, its hallmarks are sleep fragmentation, several dissociated REM-sleep/wake events (with intrusion of cataplexy, sleep-related paralyzes, and hallucinations into wake and of lucidity and enactment into REM sleep), and diurnal hypersomnolence. The latter often leads to an untimely fast transition (in less than 15 min) from wakefulness to REM sleep (sleep onset REM period: SOREMP) at nighttime (Rechtschaffen et al., 1963a), and daytime sleep (Dement et al., 1966).

The occurrence of two or more SOREMPs during the Multiple Sleep Latency Test (MSLT), which remains the most specific neurophysiological marker of NT1 disease (Richardson et al., 1978; Carskadon et al., 1986), seems of particular interest, under the assumption that DE frequencies of NT1 patients are comparable to those of healthy subjects. MSLT studies, which provide five opportunities (i.e., trials) to quantify sleep propensity and detect SOREMPs (in about two thirds of naps: Drakatos et al., 2013a,b), have already shown that DEs reported by NT1 patients after SOREMP naps are comparable in frequency (about 80%: Benbadis et al., 1995; Waihrich et al., 2006; Cipolli et al., 2020) and structural organization with DEs they report after awakening from nighttime REM sleep (Cipolli et al., 2020). The latter DEs in turn have shown to be similar to those reported by healthy subjects after late-night REM sleep (Cipolli et al., 2008; Mazzetti et al., 2010). Moreover, episodes of lucid dreaming (Fosse, 2000; Dodet et al., 2015; Rak et al., 2015) and of dream enactment (Bellucci et al., 2016) occur also in DEs of daytime SOREMP naps.

On the contrary, the available estimates of the frequency of NREM-DEs are discrepant. On the one hand, DE was reported by NT1 patients after MSLT naps with NREM sleep (hereinafter NREM naps) much less frequently (about 30%: Benbadis et al., 1995; Waihrich et al., 2006; Schinkelshoek et al., 2018) compared with not only their SOREMP naps but also nighttime and daytime NREM sleep of healthy subjects (about 60%: for review, see Nielsen, 2000, 2011b). On the other hand, in an experimental study NT1 patients reported DE with a similarly high frequency after one or two around-noon naps with NREM (about 80%) or SOREM sleep (90%) (Vogel, 1976). This discrepancy suggests that DE may be generated during daytime NREM sleep more frequently than usually reported.

To ascertain the “real” frequency of NREM-DE, here we asked NT1 patients not only to report DE (so-called “dream”) after each MSLT nap, but also, in the case of failure, to specify if they were simply unable to retrieve any content (“white dream”) or believed that no DE occurred (“no-dream”). Then, the proportions of dreams, white dreams, and no-dreams and the indicators of structural organization of DEs reported by NT1 patients after NREM naps were compared with those of DEs of individuals with subjective complaint of excessive daytime sleepiness (sc-EDS). These subjects were chosen as controls because they can take multiple daytime naps (differently of healthy subjects: Carskadon et al., 1986) without (or rarely) reaching SOREMP sleep (differently of NT1 patients: American Academy of Sleep Medicine [AASM], 2014). In this way, we attempted to estimate both the “real” frequency of DE during NREM naps and the difficulty of recalling NREM-DE contents for NT1 patients, in keeping with the general presupposition that the more complex and the longer the DE, the easier the retrieval of its contents (Foulkes and Schmidt, 1983).

## Materials and Methods

### Participants

Within the framework of a wide research program on the frequency, content, and structural characteristics of DEs in NT1 patients, we considered for the present study both the DEs of patients with final diagnosis of NT1 who had one or more MSLT naps with only NREM sleep and the DEs of sc-EDS subjects, who have almost all NREM naps.

Both NT1 patients and sc-EDS subjects were retrospectively selected among those consecutively recruited during the diagnostic procedure for suspected narcolepsy at the Narcolepsy Center of the University of Bologna from June 2018 to May 2020. They were drug free (i.e., drug naïve or after a 3 weeks discontinuation) at diagnostic workup that included the following procedures (Pizza et al., 2013): (i) clinical evaluation; (ii) subjective sleepiness assessment (Epworth Sleepiness Scale, ESS) (Vignatelli et al., 2003); (iii) 48 h continuous polysomnographic (PSG) recording (24 h for adaptation and 24 h for diagnostic purposes); (iv) five naps MSLT (Littner et al., 2005); (v) in-laboratory test to elicit cataplexy (Pizza et al., 2013; Vandi et al., 2019); and (vi) blood test and, whenever possible, lumbar puncture to search for the human leukocyte antigen (HLA) DQB1^∗^0602 allele (Mignot et al., 2006) and cerebrospinal hypocretin-1 levels, respectively. According to the current international criteria (American Academy of Sleep Medicine [AASM], 2014), a final diagnosis of NT1, narcolepsy without cataplexy (narcolepsy type 2: NT2), idiopathic hypersomnia (IH), or sc-EDS was provided.

To be eligible for the study, participants had also to have fulfilled the following criteria: (i) age between 18 and 50 years; (ii) 8 or more years of education; (iii) no history of neurological, psychiatric, or sleep comorbidity; (iv) ability to recall at least one dream per week (retrospectively evaluated over the previous 2 months); and (v) lack of global and memory-specific cognitive deficits, i.e., without scores below the cutoff points of mild deficit at Wechsler Adult Intelligence Scale—Revised (WAIS-R, Wechsler, 1981) for global and specific cognitive functions, and Wechsler Memory Scale (WMS, Wechsler, 1987) for short- and long-term memory. The 67 eligible participants were requested to report the DE developed during each MSLT nap and signed their written informed consent according to the study protocol approved by the local Ethics Committee.

#### Procedure

Before the first MSLT trial, participants were instructed that after each MSLT trial they would be asked by an investigator (C.B., blind to participants’ clinical diagnosis) to provide a report of the mental experience developed during sleep (using the classical Foiulkes (1962) instruction, “Would you tell me whatever was going through your mind before awakening?”). According to Cohen’s (1972) criteria, participants could be a) able to report contents of the previous mental experience (“dream”), b) unable to recall any content of the mental experience felt (“white dream”), or c) unable unable to recall any experience before awakening (“no dream”). In the first case, after the free (i.e., spontaneous) report, they would be asked again: “Could you remember one or more further events of the same mental experience you have just reported?” and thus could further complete their report.

#### Sleep Scoring and Analysis of Dream Reports

PSG recordings of all participants were scored by a board-certified PSG technician (S.V.) and a blind expert neurologist (L.V.) according to the international criteria (Iber et al., 2007). At the end of this analysis, 5 patients showing a comorbid sleep disorder at 48 h continuous PSG were excluded from the study.

**FIGURE 1:**
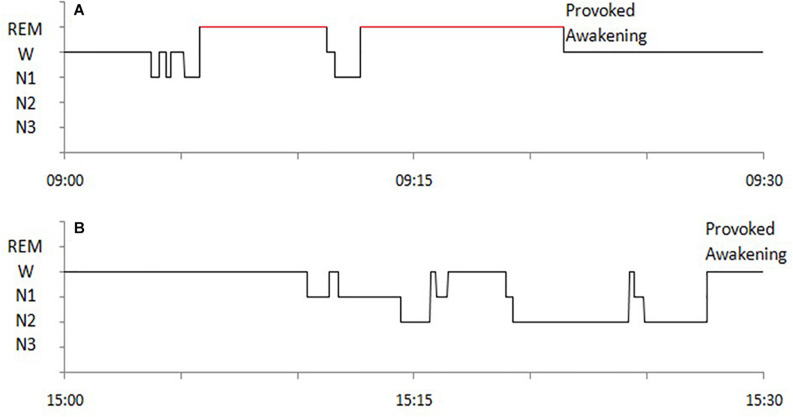
Examples of hypnograms of MSLT naps with SOREMP sleep **(A)** and with NREM sleep **(B)** of a NT1 patient.

The following sleep parameters were calculated for MSLT naps Time in bed (TBT), Total sleep time (TST), sleep latency (SL), wakefulness after sleep onset (WASO), sleep efficiency (SE), duration of each sleep stage (non-REM sleep stage 1, 2, and 3, i.e., N1, N2, N3), and occurrence of SOREMP sleep (whose duration was not considered for the study). Hypnograms of SOREMP and NREM naps are exemplified in Figure 1.

Sleep recordings and reports of NT1 patients with one or more NREM naps (17 out of 37) and of all sc-EDS subjects (*n* = 25) were considered for statistical analyses.

### Dream Recall Frequency

The proportions of dreams, white dreams, and no dreams were calculated separately out of the number of naps. The proportion of dreams informs us about the perceptual and emotional characteristics and the structural organization of NREM-DE, while the sum of the proportions of dreams and white dreams allows estimating the “real” production (as self-estimated occurrences) of DE during sleep (Fazekas et al., 2019). Indeed, the main neural EEG correlates of the sleep periods preceding dreams and white dreams are very similar, unlike those preceding no dream, for NREM sleep as well as REM sleep in healthy subjects (Siclari et al., 2017). By comparing the two measures, we attempted also to account for the previous discrepant NREM-DE estimates obtained in MSLT studies compared to the Vogel’s (1976) experimental study.

### Report Analysis

The verbatim transcripts of the whole (i.e., spontaneous plus possibly prompted) DE reports were pruned from clauses not related to or repetitive of dream contents and comments and then were scored by two expert psycholinguists (M.B. and E.R.), unaware of the study aims, of the diagnosis and type of SOREMP/NREM naps. They had to identify independently the structural organization of DEs using the rules of a story grammar (Mandler and Johnson, 1977), which allow identifying large semantic units, which are conceptual in nature, and their causal and temporal relationships (for a detailed description, see Cipolli and Poli, 1992).

**FIGURE 2:**
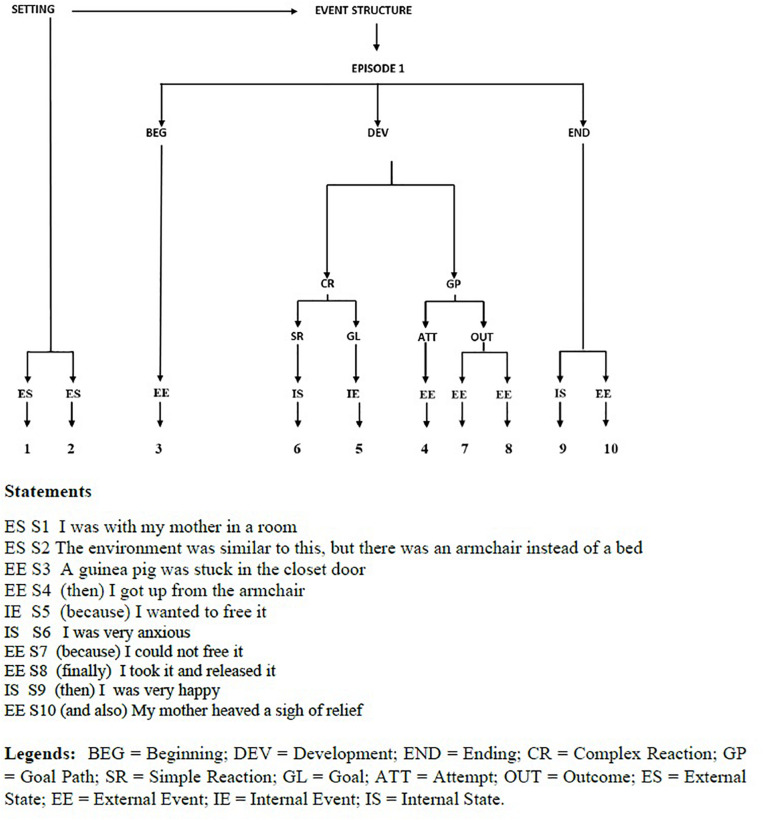
Dygraph and Statements of a dream experience reported by a NT1 patient after a nap with NREM sleep.

The outcome of story-grammar analysis is a tree structure going from the top constituent (Story, namely one or more events linked by the same Setting and Characters) to the basic nodes (Statements, describing either a State or Event). In its simplest form, a (dream-)story consists of a Setting (time and place of the event to be narrated) and an Event structure, with one or more Episodes, each having several intermediate constituents (for an example, see Figure 2).

Inter-scorer agreement was higher than 96% in parsing statements and classifying statements into intermediate constituents, 95% in classifying constituents into episodes, and complete (100%) in classifying episodes into dream-stories. The few cases of disagreement were solved through discussion between the two psycholinguists. The values of interscorer agreement were high because the story-grammar rules were explicit and not ambiguous.

The following indicators were calculated: (a) the report length (i.e., number of statements per report), (b) the number of dream-stories (i.e., sequences of connected events with the same characters and setting) per report, (c) the length of dream-stories (measured as number of statements), and (d) the indicators of structural organization of dream-stories (Cipolli et al., 1998), namely:

(d1) Context *organization* (i.e., number of statements per dream-story describing Setting), which is indicative of recall accuracy;
(d2) Sequential (i.e., temporal) *Organization* (i.e., number of statements per story realizing the actions of Event structure), which is indicative of coherence of dream contents;
(d3) Hierarchical *organization* (i.e., number of episodes per story), which is indicative of planning of dream plot.

### Data Analysis

SPSS version 21 was used for the analysis of parametric (ANOVA) and non-parametric data (χ^2^). All statistical tests were two-tailed, and alpha level was fixed at 0.05.

## Results

### Demographic, Psychometric, and PSG Data of NT1 Patients and sc-EDS Subjects

NT1 patients with NREM naps and sc-EDS subjects did not differ significantly for gender (7 male and 10 female NT1 patients vs 16 male and 9 female sc-EDS subjects: χ^2^ = 2.129, n.s.), as well as for age and years of education (see Table 1a) and values of psychometric indicators (see Table 1b).

**TABLE 1:** Values of demographic indices (1a), psychometric measures (1b), and sleep parameters (1c) of NT1 patients and sc-EDS subjects with NREM naps.

	17 NT1 patients	25 sc-EDS subjects	*F*_(1, 40)_	*p*
**1.a Demographic indices**				
Age (years)	34.5911.37	32.0010.55	0.572	0.454
Schooling (years)	12.003.02	13.122.57	1.667	0.204
**1.b Psychometric measures**				
WAIS-R verbal I.Q.	111.5911.24	105.7614.49	1.948	0.171
WAIS-R performance I.Q.	112.1812.35	107.0811.79	1.821	0.185
WAIS-R total I.Q.	113.1811.80	106.9613.73	2.316	0.136
WMS total M.Q.	111.299.40	109.8612.82	0.155	0.696
**1.c Sleep parameters**				
Time in bed (′,″)	21′0.38″6′0.42″	25.′54″2′0.44″	12.537	**0.001**
Total sleep time (′,″)	16′0.45″2′0.05″	17′0.04″3′0.52″	0.120	0.912
Sleep latency (′,″)	4′0.53″5′0.05″	8′0.50″3′0.41″	8.547	**0.01**
WASO (′,″)	1′0.08″1′0.46″	6′0.08″3′0.58″	23.601	**<0.001**
N1(′,″)	3′0.52″2′0.14″	3′0.55″1′0.51″	0.005	0.944
N2 (′,″)	10′0.11″2′0.46″	6′0.11″3′0.31″	15.400	**<0.001**
N3(′,″)	1′0.34″3′0.02″	0′0.50″1′0.21″	1.160	0.288
Sleep efficiency (%)	90.658.47	64.0720.27	33.552	**<0.001**

**NT1, narcolepsy type 1; I.Q., intelligence quotient; M.Q., memory quotient; WAIS-R, Wechsler Adult Intelligence Scale Revised; WMS, Wechsler Memory Scale; ′, minutes;″ seconds; N1, N2, N3, stage 1, 2, 3 of NREM sleep; p values in bold are statistically significant.**

Table 2 presents the occurrences of naps at MSLT trials, which were more frequent in NT1 patients than sc-EDS subjects (97.65 vs 86.40%: χ^2^ = 18.776, *p* < 0.001). The proportion of SOREMP naps was obviously much higher in NT1 patients compared with sc-EDS subjects, in whom it was negligible (66.27 vs 4.63%: χ^2^ = 82.979, *p* < 0.001).

**TABLE 2:** Occurrences of dreams, white dreams, and no dreams in NREM and SOREM naps of NT1 patients and sc-EDS subjects.

17 NT1 patients	NREM naps				SOREMP naps				No sleep
		
	T	D	WD	ND	T	D	WD	ND	
Trial 1	2	0	0	2	14	11	2	1	1
Trial 2	6	1	3	2	11	7	3	1	
Trial 3	6	3	3	0	11	6	4	1	
Trial 4	6	2	2	2	10	7	1	2	1
Trial 5	8	1	4	3	9	4	1	4	
Total *n* = 85	28	7	12	9	55	35	11	9	2
**25 sc-EDS subjects**									
Trial 1	20	16	1	3	2	2	0	0	3
Trial 2	23	6	6	11	1	0	1	0	1
Trial 3	24	11	3	10	0	0	0	0	1
Trial 4	18	6	5	7	2	0	1	1	5
Trial 5	18	6	3	9	0	0	0	0	7
Total *n* = 125	103	45	18	40	5	2	2	1	17

**T, total number of naps; D, dream; WD, white dream; ND, no dream.**

The values of sleep parameters of NREM naps of NT1 patients were significantly higher for sleep efficiency and duration of N2 sleep and lower for TIB, sleep latency, and WASO compared with those of sc-EDS subjects (see Table 1c).

### Frequency of Dream Recall

In NT1 patients, the proportion of DE reports (i.e., dreams) was significantly lower for NREM naps than for SOREMP naps (25 vs 63.64%: χ^2^ = 16.049, *p* < 0.001), while the proportion of white dreams was significantly higher (42.86 vs 20%: χ^2^ = 4.836, *p* < 0.05) (see Table 2).

The sum of the proportions of dreams and white dreams was significantly lower in NREM naps than in SOREMP naps (67.86 vs 83.64%: χ^2^ = 6.166, *p* < 0.025), while it was similar to that in NREM naps of sc-EDS subjects (61.17%: χ^2^ = 0.442, n.s.). However, the proportions of NREM naps with dreams or white dreams differed significantly in the two groups of participants (χ^2^ = 6.478, *p* < 0.02), the former being lower (25.00 vs 43.69%) and the latter being higher (42.86 vs 17.48%) in NT1 patients compared with sc-EDS subjects (see Table 2).

Table 3 presents the occurrences of dreams, white dreams, and no dreams in NREM naps of NT1 patients and sc-EDS subjects according to the sleep stage at the end of MSLT trials.

**TABLE 3:** Sleep stage or wakefulness at the end of MSLT trials resulting in NREM naps with dream, white dream, or no dream.

17 NT1 patients	NAP 1			NAP 2			NAP 3			NAP 4			NAP 5			Total			
						
	D	WD	ND	D	WD	ND	D	WD	ND	D	WD	ND	D	WD	ND	T	D	WD	ND
N1	0	0	0	0	0	0	0	1	0	0	0	0	0	0	0	1	0	1	0
N2	0	0	2	1	2	1	2	2	0	2	2	2	0	1	1	18	5	7	6
N3	0	0	0	0	1	0	1	0	0	0	1	0	1	2	1	7	2	4	1
Wakefulness	0	0	0	0	0	1	0	0	0	0	0	0	0	0	1	2	0	0	2
Total	0	0	2	1	3	2	3	3	0	2	3	2	1	3	3	28	7	12	9
**25 sc-EDS subjects**																			
N1	4	0	0	0	1	0	3	0	0	1	0	1	0	0	2	12	8	1	3
N2	6	1	2	2	4	4	3	2	4	3	2	2	4	0	2	41	18	9	14
N3	0	0	0	1	1	1	2	1	3	1	2	2	0	2	2	18	4	6	8
Wakefulness	6	0	1	3	0	6	3	0	3	1	1	2	2	1	3	32	15	2	15
Total	16	1	3	6	6	11	11	3	10	6	5	7	6	3	9	103	45	18	40

**T, total number of naps; D, dream; WD, white dream; ND, no dream.**

In NT1 patients, the end of NREM naps was more frequent in N2 sleep (64.29 vs 39.81%: χ^2^ = 5.294, *p* < 0.025) and less frequent in wakefulness compared with sc-EDS subjects (7.14 vs 31.07%: χ^2^ = 7.924, *p* < 0.01).

Table 4 presents the values of length and structural organization in SOREMP-DEs and NREM-DEs of NT1 patients and in NREM-DEs of sc-EDS subjects and the results of the one-way ANOVAs.

**TABLE 4:** Length and structural organization of DE reported by 6 NT1 patients after both NREM and SOREM naps and by 7 NT1 patients and 16 sc-EDS patients after NREM naps.

	SOREMP dreams of 6 NT1 patients with NREM and SOREMP dreams	NREM dreams of 6 NT1 patients with NREM and SOREMP dreams			NREM dreams of 7 NT1 patients	NREM dreams of 16 sc-EDS patients		
Indicators (individual mans)			**I(_1, 5)_**	**p**			***F*_(1, 21)_**	**p**
N. Statements per report (report length)	25.3614.63	8.333.27	8.280	***p* = 0.035**.	8.713.15	5.972.88	4.201	*p* = 0.053
N. Dream-stories per report	1.640.92	1.17.0.41	3.959	*p* = 0.103	1.140.38	1.200.35	0.166	p = 0.688.
N. Statements per dream-story (story length)	16.055.44	7.413.14	12.939	***p* = 0.016**	7.933.17	4.761.42	11.363	***p* = 0.003**
N. Statements in setting	3.241.44	2.331.37	2.792	*p* = 0.156	2.711.60	1.500.77	6.190	***p* = 0.021**
N. Statements in event structure	12.804.28	5.082.42	13.213	***p* = 0.015**	5.212.23	3.251.26	7.296	***p* = 0.013**
N. Episodes per dream-story	2.831.33	1.500.55	6.032	*p* = 0.058	1.430.53	1.290.43	0.452	*p* = 0.509.

*p values in bold are statistically significant.*

The comparison of DEs reported by 6 NT1 patients after both SOREMP and NREM naps showed that reports and dream-stories were significantly shorter after NREM sleep, while the number of dream-stories did not significantly differ. Moreover, dream-stories of NREM naps showed a lower sequential (significantly) and hierarchical organization (by trend) compared with dream-stories of SOREMP naps, while the contextual organization did not differ significantly.

On the contrary, NREM-DEs of 7 NT1 patients showed that both reports (by trend) and dream-stories (significantly) were longer than those of 16 sc-EDS subjects, while the number of dream-stories did not differ significantly. Moreover, dream-stories of NT1 patients showed a significantly higher contextual and sequential (but not hierarchical) organization compared with those of sc-EDS subjects.

## Discussion

The present study attempted for the first time to estimate both the “real” frequency of DE generated during MSLT naps with NREM sleep of NT1 patients by collecting not only their DE reports (dreams) but also self-evaluations of DE occurrence when they were not able to retrieve any content (white dreams), and their difficulty to retrieve contents of NREM-DE. To this aim, their frequency of dreams and white dreams and the values of the indicators of structural organization of DE reports (i.e., dreams) were compared with those of sc-EDS subjects (who likewise can take multiple NREM naps over the day: American Academy of Sleep Medicine [AASM], 2014).

Before discussing the findings obtained, it seems worth pointing out that although NREM naps occurred only in 17 out of 37 NT1 patients examined, their main neurophysiological and psychological characteristics appear reliable. Indeed, the proportions of NREM and SOREMP naps in MSLT of these 17 NT1 patients (28 and 55 out of 83 trials with naps, i.e., 33.74 and 66.26%, respectively) were consistent with those reported in the literature (Drakatos et al., 2013a,b). Moreover, the frequency of NREM naps with DE report (25%) was similar to those obtained in previous MSLT studies (about 35%, Benbadis et al., 1995; 30%, Waihrich et al., 2006; 28%, Schinkelshoek et al., 2018). Therefore, with the caution required by the small size of our sample, it can be argued that the results of the comparison of data of NT1 patients with those of sc-EDS subjects provide reliable indications on NREM-DE generation and recall. These indications are obviously more cogent for DEs generated during N2 sleep, given the longer duration of this stage and the higher proportion of NREM naps ending with it in NT1 patients compared with sc-EDS subjects.

### PSG Data

The analysis of PSG recordings showed that the sleep propensity, as expression of the homeostastic pressure, was greater and more homogeneous over the trials of NT1 patients compared with those of sc-EDS subjects, in keeping with literature data (Pizza et al., 2013). Indeed, both the proportion of failure to take a nap and the frequency of spontaneous awakening (i.e., before the end of trial) in NREM naps were significantly lower (while sleep efficiency was higher) in NT1 patients compared with sc-EDS subjects. Moreover, NREM naps of NT1 patients had not only a shorter sleep latency and a lower proportion of WASO but also a longer duration of N2 sleep (usually until the end of trial: see Table 3) compared with naps of sc-EDS subjects, which were concluded often in other NREM sleep stages or wakefulness.

### Dream Data

The values of the indicators of recall and structural organization of NREM-DEs converge to indicate that the low frequency of NREM-DEs reported by NT1 patients depends on a failure in recall after awakening rather than in generation during sleep. Indeed, while the frequency of white dreams was significantly higher in NREM naps of NT1 patients (42.86%) compared with both NREM naps of sc-EDS subjects (17.48%) and SOREMP naps of NT1 patients themselves (20%), the frequency of all NREM-DEs (i.e., dreams plus white dreams) was similar in the two groups (67.86 and 61.17%, respectively). Notably, this frequency was comparable also with those of DEs reported by healthy subjects after both daytime NREM (around 60%: for review, see Nielsen, 2011b) and nighttime NREM sleep (Pivik and Foulkes, 1968).

Moreover, the dream findings, if considered together with the PSG ones, went against the two main predictions of the arousal-retrieval model. First, the higher duration of WASO after sleep onset should lead to expectation of a higher DE recall frequency (Koulack and Goodenough, 1976; Vallat et al., 2017) in sc-EDS subjects compared with NT1 patients (in whom the value of WASO was negligible: 6′0.08″ ± 3′0.58″ vs 1′0.08″ ± 1′0.46″, respectively, see Table 1), contrary to the findings obtained. Second, the NREM naps of NT1 patients were concluded prevalently by a provoked (i.e., abrupt) awakening, which should facilitate dream recall (Goodenough et al., 1965) and, thus, lead to a higher proportion of dreams compared with white dreams in NT1 patients than sc-EDS subjects, again contrary to our findings.

Finally, also the values of the indicators of length and structural organization of NREM-DEs (lower than those of SOREM-DEs of NT1 patients but higher than those of NREM-DEs of sc-EDS subjects: see Table 4) were coherent with the hypothesis of a recall failure, under the assumption that recall is easier for more complex and long DEs (Foulkes and Schmidt, 1983). Indeed, NT1 patients are used to retrieve a wealth of vivid and bizarre DE contents after daytime SOREMP as well as after nighttime REM sleep (Broughton, 1982; Fosse, 2000). Notably, as shown by studies on healthy subjects, both vividness (Salzarulo and Cipolli, 1979; Foulkes and Schmidt, 1983) and bizarreness of DE contents (Cipolli et al., 1993; Nielsen, 2011a) facilitate their retrieval after awakening These features of DE contents clearly concur to determine the positive relationship between the quality of DE contents (according to the definition of this notion proposed by Fazekas et al., 2019) and the effectiveness of recall. The specific role played by these features in the general process of DE recall might be established in studies carried out on participants undergone some preliminary training (see below).

Explaining the failure in recall (i.e., the discrepancy between the occurrence and reporting) of NREM-DE in terms of a negative attitude of NT1 patients toward recall of short and poorly structured DEs appears plausible also on the basis of the historical evolution of the estimates of DE frequency. Indeed, the proportions of DEs recalled have been enhanced over years from the early estimates (Dement and Kleitman, 1957a,b) to the normative ones (see Nielsen, 2000) by interviewing participants (a) using a more extensive definition of the notion of “dream” (including any mental experience during sleep), (b) after a preliminary session for adaptation to laboratory content and task of dream recall (for review, see Cohen, 1979), and (c) using articulated questions to guide participants in retrieving features of DE relevant for the study aim and design (Kahn et al., 2000). It is apparent that first-diagnosed NT1 patients interviewed after MSLT naps had not any previous experience of dream reporting in laboratory context. It seems thus plausible that they are likely to desist from attempting to retrieve the less vivid and bizarre contents as usually the NREM-sleep ones are compared with those they are used to recall effortlessly after awakening from (SO)REM sleep (Broughton, 1982; Fosse, 2000).

Also, the findings of two recent studies speak in favor of this explanation. The former study showed that a low frequency of dream recall (25.64%) is obtained by administering a dream questionnaire after NT1 patients got up on their own (Schredl and Olbrich, 2019). This low recall frequency (albeit not distinguishing NREM-DEs from SOREMP-DEs) confirms that untrained NT1 patients estimate to have had a DE when some contents are easily accessible on delayed (after some hours) as well as on immediate recall (as in the present study). The latter study, carried out using a within-subject design and estimating DE recall of NT patients in terms of subjective evaluation (yes/no), showed that a lower delta power over parietal and centro-parietal areas is predictive of successful (i.e., yes) recall for DE of both NREM and REM naps (D’Atri et al., 2019). The large overlapping of the EEG correlates of successful recall after SOREMP and NREM naps suggests that REM and NREM sleep share a similar machinery for dream recall in NT1 patients as well as in healthy subjects (Siclari et al., 2017). This finding, in weakening the alternative hypothesis of an intrinsically lower capacity of NREM sleep of NT1 patients for memory encoding of DE contents, further corroborates the interpretative hypothesis put forward here that a negative attitude toward recall is responsible of the lower frequency of NREM-DEs reported compared the generated ones.

Notably, also the residual difference (about 15%) between the present estimate of the “real” NREM-DE frequency (as sum of dreams and white dreams) and that of Vogel’s (1976) experimental study (i.e., with planned awakening and immediate DE recall) on NT1 patients may be accounted for in keeping with the above hypothesis. Indeed, Vogel’s patients not only were under a greater sleep pressure at the moment of one or two around-noon naps compared with patients undergone MSLT trial every, 2 h but also underwent a preliminary training for DE recall. As shown by studies on healthy subjects, a great sleep pressure enhances such dreamlike features as vividness and bizarreness of DE contents after daytime (Carr and Nielsen, 2015) as well as nighttime NREM sleep (Nielsen et al., 2005), and a preliminary training enhances the rate of successful recall of specific features of dream content (Kahn et al., 2000).

It appears thus plausible that some preliminary training during the diagnostic procedure (for example, after the first night of PSG recordings), by modifying the negative attitude toward recall of less vivid and bizarre DE contents in NT1 patients undergone MSLT, can enhance the proportion of DEs reported (i.e., dreams) after NREM as well as SOREMP naps (their proportions of white dreams in the present study being 42.86 and 20%, respectively, see Table 2). Moreover, the D’Atri et al. (2019) study showed a large overlapping of EEG correlates of successful recall after SOREMP and NREM naps of NT1 patients. Therefore, gathering this item of evidence in future studies would concur to confirm that MSLT may be used as a parsimonious (being within the clinical routine) and extended (from mid-morning to late afternoon) multiple-nap protocol to investigate the characteristics of content and structural organization of NREM-DEs as well as of SOREMP-DEs (Cipolli et al., 2020). Obviously MSLT studies, given the intrinsic limitation of the fixed nap duration, cannot substitute completely the experimental ones but may provide useful insights into the general process of DE generation.

### Future Directions

Collecting an amount of DE reports fairly comparable to that of DEs generated by NT1 patients during NREM sleep (namely, in about two thirds of NREM naps) would allow not only to identify (by using spectral analysis techniques) the global and local EEG correlates of DE recall but also cast light on DE generation as a continuum across sleep stages. This issue has been recently pointed out (Nielsen, 2017) as at least crucial as that of the wake–sleep continuity (Schredl and Hofmann, 2003) for the understanding of the relationships between the neurophysiological EEG signals and the functioning of the cognitive processes underlying DE generation. As shown by studies on healthy subjects, the contents of DEs result from the often repeated activation of more or less recent memories and some rebinding of their features in subsequent sleep stages. This iterative processing is indicated by both the interrelated (i.e., similar) contents present in NREM- and REM-DEs of the same night (Rechtschaffen et al., 1963b; Cipolli et al., 1988) and the repeated incorporation of pre-sleep stimuli as contents into DEs of subsequent sleep stages (Wamsley et al., 2010).

Moreover, the prevalent but not exclusive transition from N1 to SOREMP sleep (Drakatos et al., 2013a; Cipolli et al., 2020) can offer the opportunity to observe two potentially different types of across-stage continuity by comparing the content and structural organization of DEs reported after MSLT naps in which SOREMP has been preceded by only N1 or also N2 sleep. Since frontal theta activity increases from the first to the last 30 s of N1 (as shown in healthy subjects: Picchioni et al., 2008), this comparison would clarify whether the complexity of the story-like plot of SOREMP-DEs is facilitated by the continuity with the high imagery typical of N1 (i.e., the construction of a mental fictive scenario, with spatial location of the imagined event: Hori et al., 1994; Stenstrom et al., 2012) or by the spreading of associative networks typical of N2 (as indicated by spindle density: Studte et al., 2015; Sopp et al., 2018).

These indications might be complemented by those gathered using properly experimental protocols (to be applied on voluntary NT1 patients after diagnostic routine) adequate to establish how pre-sleep stimuli or new tasks are incorporated as contents into the DEs of subsequent naps with only N1, N1 plus N2 sleep, or also SOREMP sleep. The variations in the rate and modality of incorporations according to the stage of prior sleep may shed new light on the role played in particular by N1 in the process of sleep-dependent memory consolidation not only per se (as already shown on healthy subjects: Lahl et al., 2008; Wamsley et al., 2010), but also in interaction with SOREMP sleep (which is only observable on NT1 patients).

## Conclusion

This study ascertained that the frequency of DEs generated during NREM naps of NT1 patients at MSLT is higher than usually estimated and fully comparable to those both of sc-EDS subjects and (even more importantly) of healthy subjects. Moreover, the structural organization of the reported NREM-DEs indicated that the discrepancy between the frequencies of DEs generated and reported depends on the attitude toward recall of NT1 patients and, thus, can be reversed by some training before MSLT trials. Given the peculiar neurophysiology of sleep in NT1 patients, enhancing the frequency and accuracy of their DE reports after NREM naps may provide new insights into some issues (such as the across-stages continuity in the functioning of the cognitive processes underlying DE generation) the approach to which is harder in healthy subjects.

## Data Availability Statement

The raw data supporting the conclusions of this article will be made available by the authors, without undue reservation.

## Ethics Statement

The studies involving human participants were reviewed and approved by the Ethics Committee of the University of Bologna. The participants provided their written informed consent to participate in this study.

## Author Contributions

CC, FP, MM, and GP conceived and designed the study. CB collected the dream data. GT and SV collected polysomnographic data. MM and CB carried outs statistical analysis. CC and FP wrote the first draft. All the authors approved the final version of the manuscript after the changes they suggested.

## Conflict of Interest

The authors declare that the research was conducted in the absence of any commercial or financial relationships that could be construed as a potential conflict of interest.
